# Methanolic Extract of *Cimicifuga foetida* Induces G_1_ Cell Cycle Arrest and Apoptosis and Inhibits Metastasis of Glioma Cells

**DOI:** 10.3390/nu16193254

**Published:** 2024-09-26

**Authors:** Chih-Hsuan Chang, Hung-Pei Tsai, Ming-Hong Yen, Chien-Ju Lin

**Affiliations:** 1 School of Pharmacy, College of Pharmacy, Kaohsiung Medical University, Kaohsiung 80756, Taiwan; sugar0306@gmail.com (C.-H.C.); yen@gap.kmu.edu.tw (M.-H.Y.); 2 Division of Neurosurgery, Department of Surgery, Kaohsiung Medical University Hospital, Kaohsiung 80708, Taiwan; carbugino@gmail.com

**Keywords:** *Cimicifuga foetida*, glioma, cell cycle arrest, apoptosis, metastasis inhibition

## Abstract

Background: Glioblastoma multiforme (GBM) is among the most aggressive and challenging brain tumors, with limited treatment options. *Cimicifuga foetida*, a traditional Chinese medicine, has shown promise due to its bioactive components. This study investigates the anti-glioma effects of a methanolic extract of *C. foetida* (CF-ME) in GBM cell lines. Methods: The effects of CF-ME and its index compounds (caffeic acid, cimifugin, ferulic acid, and isoferulic acid) on GBM cell viability were assessed using MTT assays on U87 MG, A172, and T98G cell lines. The ability of CF-ME to induce cell cycle arrest, apoptosis, and autophagy and inhibit metastasis was evaluated using flow cytometry, Western blotting, and functional assays. Additionally, the synergistic potential of CF-ME with temozolomide (TMZ) was explored. Results: CF-ME significantly reduced GBM cell viability in a dose- and time-dependent manner, induced G1 phase cell cycle arrest, promoted apoptosis via caspase activation, and triggered autophagy. CF-ME also inhibited GBM cell invasion, migration, and adhesion, likely by modulating epithelial–mesenchymal transition (EMT) markers. Combined with TMZ, CF-ME further enhanced reduced GBM cell viability, suggesting a potential synergistic effect. However, the individual index compounds of CF-ME exhibited only modest inhibitory effects, indicating that the full anti-glioma activity may result from the synergistic interactions among its components. Conclusions: CF-ME exhibited potent anti-glioma activity through multiple mechanisms, including cell cycle arrest, apoptosis, autophagy, and the inhibition of metastasis. Combining CF-ME with TMZ further enhanced its therapeutic potential, making it a promising candidate for adjuvant therapy in glioblastoma treatment.

## 1. Introduction

Glioblastoma multiforme (GBM) is the most common and aggressive type of malignant glioma in adults, classified as a Grade IV astrocytoma by the World Health Organization (WHO) [1]. Malignant gliomas, including astrocytomas, oligodendrogliomas, and oligoastrocytomas, are the primary malignant brain tumors with the highest incidence rates [2]. GBM accounts for 50.1% of all malignant brain tumors, exhibiting high mobility and invasiveness and contributing to poor prognosis [3]. The 1-year survival rate for GBM patients is approximately 35%, and the 5-year survival rate is <5% [4]. Standard GBM treatment involves a combination of surgery, radiotherapy, and chemotherapy [5]. However, despite advances in treatment, GBM remains a highly lethal cancer due to its aggressive nature and the difficulty of delivering drugs effectively across the blood–brain barrier (BBB). Temozolomide (TMZ), the primary chemotherapeutic agent for GBM, can cross the BBB and has shown effectiveness in treating gliomas [6]. TMZ functions as an alkylating agent, methylating DNA and ultimately inducing cancer cell death [7]. However, despite its widespread use, TMZ is associated with several adverse side effects, including nausea, fatigue, hepatotoxicity, and hematological toxicity, which can severely affect patient quality of life and treatment adherence [8]. Moreover, the development of resistance to both radiotherapy and chemotherapy, including TMZ, is a growing concern in GBM treatment, further complicating the management of the disease. These challenges underscore the need for novel therapeutic strategies that can overcome drug resistance, enhance treatment efficacy, and minimize side effects, highlighting the potential role of natural compounds in cancer therapy.

*Cimicifugae rhizoma*, known as Sheng Ma in traditional Chinese medicine (TCM), is derived from the dried rhizomes of *Cimicifuga foetida*, *C. heracleifolia*, and *C. dahurica*, all members of the *Ranunculaceae* family [9]. According to the Taiwan Herbal Pharmacopeia, *C. rhizoma* typically appears as irregular masses, measuring approximately 10–20 cm in length and 2–4 cm in diameter. The surface is blackish-brown, with hollow spaces and reticular furrows. It has a hard, light texture and is difficult to break, with an uneven fracture [9]. In TCM, *Cimicifugae rhizoma* is classified as mildly cold, with a pungent and sweet flavor. It targets the meridians of the lungs, spleen, stomach, and large intestine. Traditionally, it is used to relieve exterior disorders and is traditionally used to treat rashes, headaches, and sore throats [10]. Modern pharmacological studies have identified its antiviral [11,12], anti-inflammatory [13], immunomodulatory [14], and anti-tumor activities [15,16,17]. Research has demonstrated that ethanol extracts of *C. rhizoma*, as well as isolated compounds, such as triterpenoids and actein, can suppress various cancers, including liver, breast, and lung cancers, through mechanisms such as inducing cell cycle arrest, promoting apoptosis, and inhibiting cell metastasis [18,19,20]. Despite the promising therapeutic potential of Cimicifuga extracts, their application in brain cancer, specifically GBM, remains largely unexplored. Given the urgent need for novel treatments in GBM and the growing interest in natural compounds for cancer therapy, this study aims to investigate the anti-glioma effects of a methanolic extract of *C. foetida* (CF-ME) on GBM cell lines. By examining CF-ME’s impact on cell viability, cell cycle progression, apoptosis, autophagy, and metastasis, we seek to elucidate its potential as an adjuvant therapy for GBM. Our findings suggest that CF-ME exhibits potent anti-glioma activity through multiple mechanisms and may enhance the efficacy of current treatments when used in combination.

## 2. Materials and Methods

### 2.1. Materials

#### 2.1.1. Source and Extraction of *C. rhizoma*

The *C. rhizoma* material used in this study was purchased from the Hung Chuan Chinese Medicine Store (Sanmin District, Kaohsiung City, Taiwan). The materials were sourced from JET TURN Pharmaceutical Technology (Kaohsiung, Taiwan). The specimens were identified by Dr. Ming-Hong Yen, a specialist in traditional Chinese herbal medicine. The specimens were chopped into small pieces, oven-dried, and weighed. The dried material was soaked in 10 times its weight of water or methanol, subjected to 1 h of ultrasonic shock, and left for 1 d to obtain the first filtrate. This process was repeated with fresh water or methanol to obtain a second filtrate. The filtrates were mixed and concentrated using a rotary evaporator (Vacuum Controller VC-7600, Panchum, Kaohsiung, Taiwan) and freeze dryer (FDU-2000, EYELA, Tokyo, Japan) to obtain a dry powder of the extract, which was stored at −20 °C. Stock solutions were prepared with sterile water (for the water extract) or dimethyl sulfoxide (DMSO) (for the methanolic extract) for subsequent experiments.

#### 2.1.2. HPLC Identification of the *C. rhizoma* Extract

To prepare the samples, methanol was used to prepare 10 mg/mL CF-ME and individual solutions of caffeic acid, cimifugin, ferulic acid, and isoferulic acid at concentrations of 3.125, 6.25, 12.5, 15.625, and 25 μg/mL. These samples were analyzed via high-performance liquid chromatography (HPLC) with ultraviolet detection using a Hitachi L-7100 pump, Hitachi L-7200 autosampler, and Hitachi 5420 UV-vis detector (HITACHI, Tokyo, Japan). Chromatography was performed on a Mightysil RP-18 GP column (5 μm, 4.6 × 250 mm), with a flow rate of 1.0 mL/min and an injection volume of 20 μL. The UV detection wavelength was set at 254 nm. The mobile phases used were (A) 0.1% H_3_PO_4_ and (B) acetonitrile. The condition of gradient elution was applied as follows: 0–5 min, 92% → 82% A; 5–20 min, 82% → 76% A; 20–32 min, 76% → 66% A; 32–40 min, 66% → 45% A; 40–48 min, 45% → 30% A; 48–54 min, 30% → 30% A; 54–56 min, 30% → 60% A; 56–58 min, 60% → 92% A; 58–68 min, 92% A. Acetaminophen was used as the internal control.

#### 2.1.3. Cell Lines and Cell Culture

U87 MG, A172, and T98G (GBM cell lines) and SVGp12 (normal human glial cell line) were obtained from the Bioresource Collection and Research Center (BCRC). U87 MG and A172 cells were cultured in Dulbecco’s Modified Eagle Medium (DMEM), T98G cells in Minimum Essential Medium (MEM), and SVGp12 cells in Minimum Essential Medium-α (MEM-α). All media were supplemented with 10% fetal bovine serum (FBS), 100 units/mL penicillin, 100 μg/mL streptomycin, and 0.25 μg/mL amphotericin B (Pen-Strep-AmphoB solution, Sartorius, Göttingen, Germany). MEM also contained 1 mM sodium pyruvate and 1 mM non-essential amino acids. Media and supplements were sourced from Hyclone (Vancouver, Canada). All cells were maintained in a cell culture incubator (Thermo Electron Corporation; Waltham, MA, USA) with 5% CO_2_ at 37 °C.

### 2.2. Methods

#### 2.2.1. Cell Viability Assay

Cell viability was determined using the 3-(4,5-dimethylthiazol-2-yl)-2,5-diphenyltetrazolium bromide (MTT) assay. After drug treatment, cells were incubated with 0.5 mg/mL MTT solution for 1 h, and the formazan was dissolved in DMSO. Absorbance was measured with an ELISA reader (Bio-Tek Instruments Inc., Winooski, VT, USA) at a wavelength of 540 nm, and the percentage of cell viability was calculated.

#### 2.2.2. Cell Cycle Analysis

Cells were seeded on 6-well plates (1.5 × 10^5^ cells/well) and treated with 0–150 μg/mL CF-ME for 72 h or 100 μg/mL CF-ME for 24, 48, and 72 h. Cells were collected and fixed in 75% alcohol at 4 °C for 16 h. After centrifugation, cells were washed with phosphate-buffered saline (PBS) and treated with RNase A for 30 min, followed by staining with propidium iodide (PI) for 20 min at 37 °C. The cell cycle was analyzed using flow cytometry and CXP software V2.3 (Beckman Coulter, Brea, CA, USA).

#### 2.2.3. Cell Apoptosis Analysis

Cells were seeded on 6-well plates (1.5 × 10^5^ cells/well) and treated with 0–150 μg/mL CF-ME for 72 h or 100 μg/mL CF-ME for 24, 48, and 72 h. Cells were collected in HEPES solution and incubated with annexin V-FITC and PI for 20 min. Apoptosis was detected via flow cytometry and analyzed using CXP software (Beckman Coulter, Brea, CA, USA). The lower left, upper left, lower right, and upper right quadrants represent the percentages of normal, necrotic, early, and late apoptotic cells, respectively. Total apoptosis was calculated as the sum of early and late apoptosis.

#### 2.2.4. Caspase Activity Assays

After drug treatment, protein samples were collected by lysing the cells with radio-immunoprecipitation assay (RIPA) buffer. Using bovine serum albumin (BSA) as a standard, protein concentrations were determined using the bicinchoninic acid (BCA) assay by measuring the absorbance at a wavelength of 562 nm. Caspase 3, 8, and 9 activities were assayed using Caspase Colorimetric Activity Assay Kits (Millipore, Billerica, MA, USA) according to the manufacturer’s instructions. Protein samples were incubated with caspase substrate for 1–2 h at 37 °C. Absorbance was measured at 405 nm using an ELISA reader, and caspase activity was expressed as fold change compared with the control group.

#### 2.2.5. Cell Autophagy Analysis

The formation of acidic vesicular organelles (AVOs), the characteristic of autophagy, could be analyzed by flow cytometry with acridine orange stain. Cells were seeded on 6-well plates (1.5 × 10^5^ cells/well) and treated with 0–150 μg/mL CF-ME for 72 h or 100 μg/mL CF-ME for 24, 48, and 72 h. Cells were collected in PBS solution and incubated with acridine orange for 15 min at 37 °C. The formation of AVOs was detected via flow cytometry and analyzed using CXP software (Beckman Coulter, Brea, CA, USA). The sum of the upper left and upper right quadrants represents the percentages of autophagic cells.

#### 2.2.6. Migration Assay

Cell migration was evaluated using a wound-healing assay. Cells were seeded on 12-well plates (3 × 10^5^ cells/well) and cultured for 24 h. Wounds were created by scratching cells with a sterile 10 µL tip. Cells were treated with CF-ME after washing with PBS. Wound closure was photographed under an inverted microscope (SDPTOP, Ningbo, China) at 0, 6, 12, and 24 h after treatment. Wound width was measured using the MShot Image Analysis System 1.5.2.

#### 2.2.7. Invasion Assay

The in vitro invasion assay was conducted using SPLInsert™ Hanging plates (SPL Life Sciences, Pocheon-si, Gyeonggi-do, Republic of Korea). CF-ME-treated U87 MG, A172, and T98G cells were seeded at a density of 1 × 10^4^ cells/Transwell insert in serum-free medium in the upper chamber, and serum-containing culture medium was added to the lower chamber. After 24 h, the cells were washed with PBS, fixed with 4% formaldehyde for 15 min, and stained with 0.5% crystal violet solution. Cells in the upper layer of the insert were carefully removed using cotton swabs, while cells that invaded through the insert were photographed and counted under an inverted microscope (SDPTOP, Ningbo, China). Cell numbers were counted in three randomly selected fields per insert.

#### 2.2.8. Cell Adhesion Assay

U87 MG, A172, and T98G cells were cultured in 6-well plates at a density of 1.5 × 10^5^ cells/well and treated with 0–150 μg/mL CF-ME for 72 h. After treatment, the cells were harvested via trypsinization and re-seeded in 12-well plates at a density of 1 × 10^4^ cells/well in triplicate wells. After 1 and 24 h of incubation, non-adherent cells were removed by washing with PBS. Adherent cells were fixed with 4% formaldehyde for 15 min, stained with 0.5% crystal violet solution, and photographed using an inverted microscope (SDPTOP; Ningbo, China). Six random regions/well were selected to count the number of cells, and the relative percentage of adherent cells was calculated.

#### 2.2.9. Western Blotting

After drug treatment, the cells were lysed with RIPA buffer, and protein samples were collected. Protein concentration was determined using the BCA assay. An equal amount of protein (50 μg) from each group was separated via sodium dodecyl sulfate-polyacrylamide gel electrophoresis (SDS-PAGE) and transferred to polyvinylidene fluoride (PVDF) membranes (Cytiva/Hyclone, Vancouver, BC, Canada). The membranes were blocked with 5% skim milk in TBS-T buffer (11 mM Tris-base pH 7.4, 154 mM NaCl, 0.1% Tween-20) for 1 h and incubated with specific primary antibodies for 16 h at 4 °C. Subsequently, the membranes were incubated with the appropriate anti-mouse or anti-rabbit secondary antibodies at room temperature for 1–2 h. Immunoreactive bands were detected using enhanced chemiluminescence (ECL) (Millipore, Billerica, MA, USA) and recorded with a MultiGel-21 Image System (TOP BIO CO., New Taipei City, Taiwan). The density of the bands was determined using ImageJ software 1.51j8 and normalized to β-actin. The antibodies used included anti-GADD45A, p21, CDK6, caspase 3, cleaved caspase 3, poly (ADP-ribose) polymerase (PARP), and horseradish peroxidase (HRP)-conjugated goat anti-mouse and anti-rabbit immunoglobulin G (IgG) purchased from Cell Signaling Technology (Beverly, MA, USA). Antibodies against vimentin, N-cadherin, E-cadherin, and cyclin D1 were purchased from Proteintech (Chicago, IL, USA). Anti-β-actin antibody was purchased from Millipore (Billerica, MA, USA).

#### 2.2.10. Statistical Analysis

At least three independent experiments were conducted for each assay, and the data are expressed as mean ± standard deviation (SD). Statistical analysis was performed using one-way analysis of variance (ANOVA) to compare three or more groups. A *p*-value of <0.05 was considered statistically significant.

## 3. Results

### 3.1. Investigation of the Effects of C. foetida Extracts on Glioma Cell Growth

**Figure 1:**
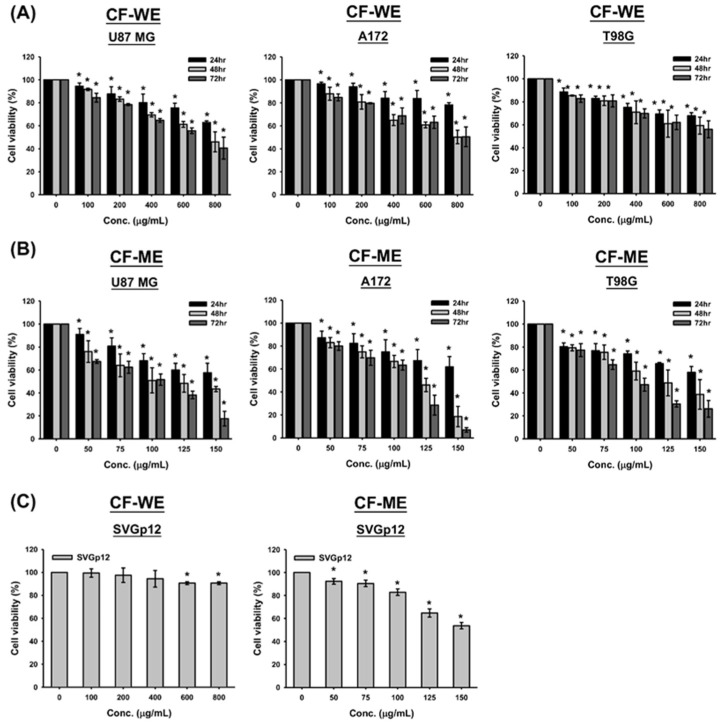
Effects of water (CF-WE) and methanol (CF-ME) extracts of *Cimicifuga foetida* on the viability of glioblastoma (GBM) and glial cells. (**A**) Cell viability of U87 MG, A172, and T98G GBM cell lines treated with varying concentrations (0–800 μg/mL) of CF-WE for 24, 48, and 72 h showed a significant dose- and time-dependent decrease in cell viability, with stronger effects at higher concentrations and longer treatment durations. (**B**) Cell viability of U87 MG, A172, and T98G GBM cell lines treated with CF-ME (0–150 μg/mL) for the same durations exhibited a stronger inhibitory effect, also in a dose- and time-dependent manner. (**C**) Cell viability of human normal glial cell line SVGp12 treated with CF-WE (left) and CF-ME (right) under similar conditions showed reduced cell viability, but SVGp12 cells exhibited higher overall viability and IC_50_ values than GBM cells, indicating lower toxicity toward normal glial cells. * Asterisks indicate statistical significance compared to control (0 μg/mL) (*p* < 0.05). Error bars represent the mean ± SD of three independent experiments.

This study evaluates the effects of water (CF-WE) and methanol extracts (CF-ME) of *C. foetida* on glioma cell growth and viability. The MTT assay was used to assess short-term cell viability, while the colony formation assay evaluated long-term viability. U87 MG, A172, and T98G glioma cell lines were treated with CF-WE at concentrations ranging from 0 to 800 μg/mL and CF-ME at concentrations from 0 to 150 μg/mL for 24, 48, and 72 h, respectively. Treatment revealed a significant decrease in cell viability, and these changes were more pronounced following CF-ME treatment (Figure 1A,B).

In addition, both CF-WE and CF-ME treatments resulted in dose- and time-dependent reductions in cell viability across all GBM cell lines tested (Figure 1A,B). Specifically, after 72 h of CF-WE treatment, the IC_50_ value was 662.5 ± 70.0 μg/mL for U87 MG cells, while the IC_50_ values for A172 and T98G cells exceeded 800 μg/mL. In contrast, CF-ME demonstrated a stronger inhibitory effect, with IC_50_ values of 94.3 ± 5.2 μg/mL for U87 MG, 96.5 ± 3.7 μg/mL for A172, and 97.3 ± 1.6 μg/mL for T98G cells after 72 h of exposure (Table 1). To assess the selectivity and toxicity of these extracts, the normal human glial cell line SVGp12 was also treated with CF-WE and CF-ME under similar conditions. The results indicated that SVGp12 cells exhibited higher viability and IC_50_ values than the glioma cells (Figure 1C and Table 1). These findings suggest that both CF-WE and CF-ME effectively reduced glioma cell viability while exhibiting lower toxicity toward normal glial cells, with CF-ME showing superior efficacy. Consequently, CF-ME at 100 μg/mL was selected for subsequent experiments. These data support the idea that both water and methanol extracts of *C. foetida* suppress glioma cell growth and reduce cell viability. The methanol extract, in particular, showed greater efficacy and lower toxicity toward normal cells, making it a promising candidate for further investigation in glioma treatment strategies.

### 3.2. CF-ME Induces G_1_ Phase Cell Cycle Arrest in Glioma Cells

**Figure 2:**
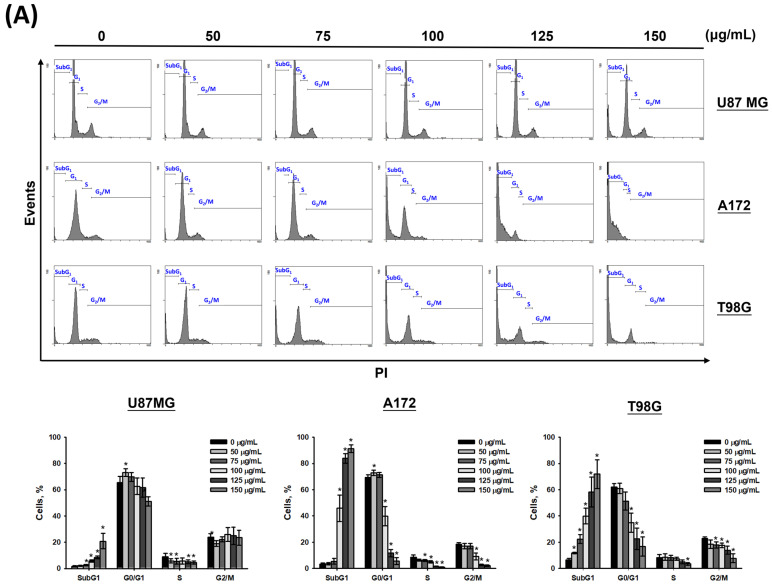

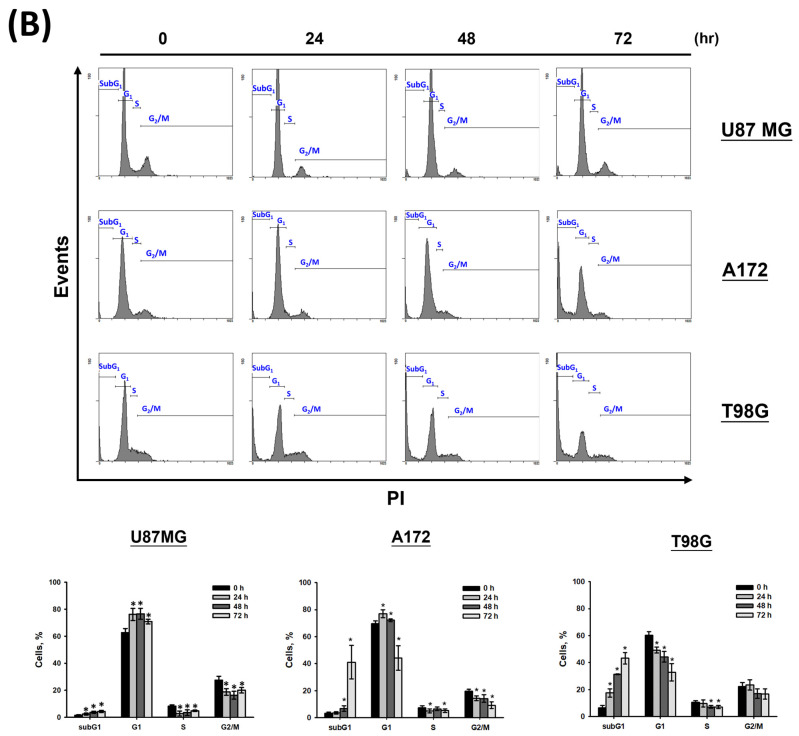

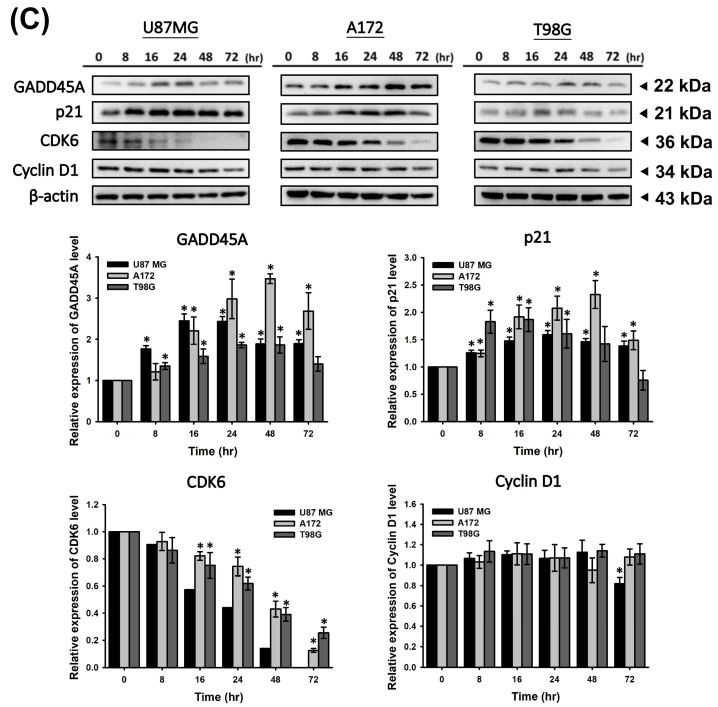
CF-ME induces G_1_ phase cell cycle arrest in glioblastoma (GBM) cells. (**A**) Flow cytometry analysis of U87 MG, A172, and T98G GBM cell lines treated with CF-ME (0–150 μg/mL) for 72 h. The histograms show the distribution of cells in different phases of the cell cycle (sub-G_1_, G_0_/G_1_, S, G_2_/M), indicating a dose-dependent increase in G_1_ phase arrest and a corresponding decrease in S and G_2_/M phases across all cell lines. (**B**) Flow cytometry analysis of U87 MG, A172, and T98G cells treated with 100 μg/mL CF-ME for 24, 48, and 72 h display a temporal progression of cell cycle arrest, with a significant accumulation of cells in the G_1_ phase over time, and an elevation in the sub-G_1_ phase, indicative of apoptosis. (**C**) Western blot analysis of GADD45A, p21, CDK6, and cyclin D1 in U87 MG, A172, and T98G cells treated with 100 μg/mL CF-ME for 0–72 h. The graphs below depict the relative protein expression levels normalized to β-actin. The results show a time-dependent upregulation of GADD45A and p21, downregulation of CDK6, and minimal changes in cyclin D1 levels, suggesting that CF-ME induces G_1_ phase cell cycle arrest through the GADD45A/p21/CDK6 signaling pathway. * Asterisks indicate statistical significance compared to control (0 μg/mL or 0 h) (*p* < 0.05). Error bars represent the mean ± SD of three independent experiments.

To determine whether CF-ME induces cell cycle arrest and suppresses cell proliferation, U87 MG, A172, and T98G glioma cells were treated with varying concentrations (0–150 μg/mL) of CF-ME for 72 h (Figure 2A) or with 100 μg/mL CF-ME for 24, 48, and 72 h (Figure 2B). Cell cycle analysis was performed using flow cytometry with PI staining. The results indicated that CF-ME induced G_1_ phase cell cycle arrest in glioma cells at lower concentrations and shorter exposure times (Figure 2A,B). In contrast, higher doses and prolonged exposure increased the number of cells in the sub-G1 phase, indicative of apoptosis. Further investigations focused on the molecular mechanisms underlying G_1_ phase arrest. Specifically, the study examined the expression of cell cycle-related proteins, such as CDK6 and cyclin D1, as well as the common cell cycle regulators p21 and GADD45A. U87 MG, A172, and T98G cells were treated with 100 μg/mL CF-ME for varying durations (8, 16, 24, 48, and 72 h), and protein levels were analyzed via Western blotting (Figure 2C). The results revealed an increase in GADD45A and p21 protein levels after 8–16 h of treatment, while CDK6 expression decreased in a time-dependent manner. Only minor changes in the cyclin D1 levels were observed (Figure 2C). These findings suggest that CF-ME induces G1 phase cell cycle arrest in glioma cells via the GADD45A/p21/CDK6 signaling pathway.

### 3.3. CF-ME Induces Apoptosis in Glioma Cells

**Figure 3:**
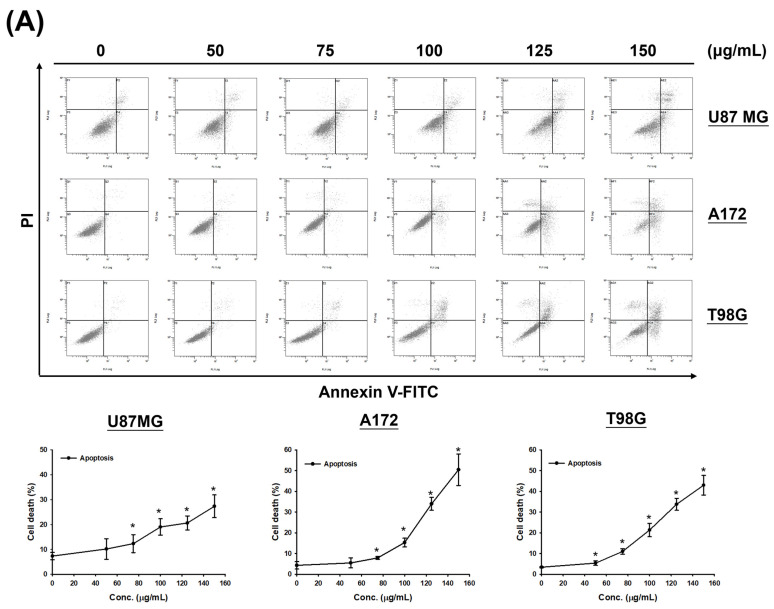

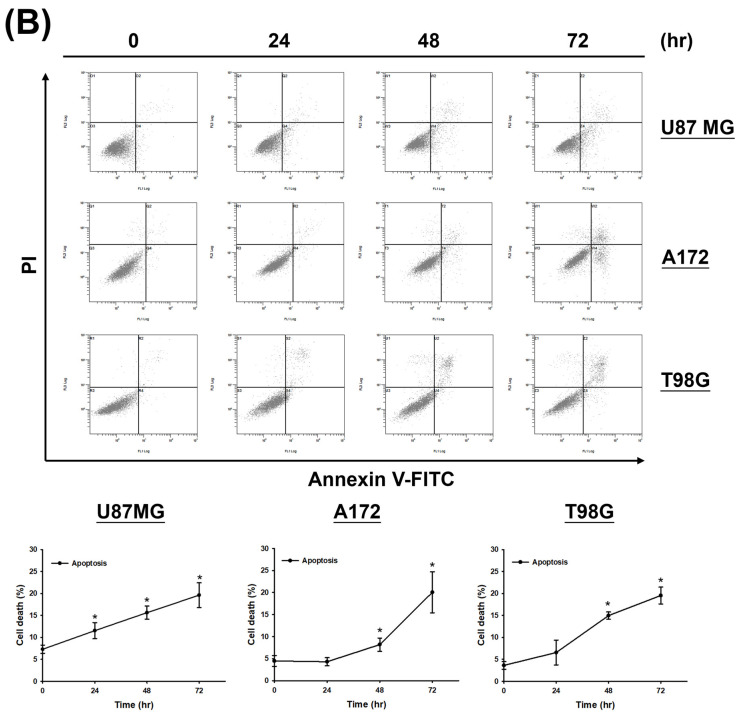

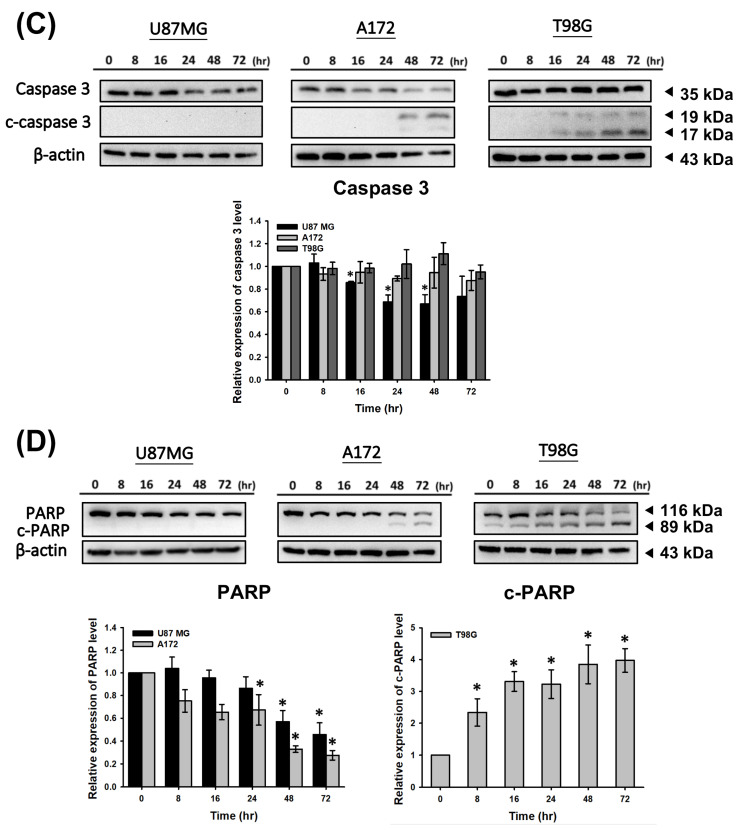
CF-ME induces apoptosis in GBM cells. (**A**) Flow cytometry analysis of apoptosis in U87 MG, A172, and T98G GBM cell lines treated with CF-ME (0–150 μg/mL) for 72 h show a dose-dependent increase in apoptosis. Cells were stained with Annexin V-FITC and PI; the dot plots distinguish early apoptotic (Annexin V-positive, PI-negative) from late apoptotic/necrotic cells (Annexin V-positive, PI-positive). The graphs below show a dose-dependent increase in the percentage of apoptotic cells across all GBM cell lines. (**B**) Time-course analysis of apoptosis in U87 MG, A172, and T98G cells treated with 100 μg/mL CF-ME for 24, 48, and 72 h show a time-dependent increase in both early and late apoptotic cells over time. (**C**) Western blot analysis of caspase 3 and cleaved caspase 3 (c-caspase 3) in U87 MG, A172, and T98G cells treated with 100 μg/mL CF-ME for 0–72 h. The accompanying graph shows the relative expression levels of c-caspase 3 normalized to β-actin. CF-ME treatment showed increased c-caspase 3 levels in A172 and T98G cells, with a decrease in U87 MG cells, suggesting the activation of the caspase pathway in apoptosis. (**D**) Western blot analysis of poly (ADP-ribose) polymerase (PARP) and cleaved PARP (c-PARP) in U87 MG, A172, and T98G cells treated with 100 μg/mL CF-ME for 0–72 h. The graphs below illustrate the relative expression levels of c-PARP and total PARP normalized to β-actin. CF-ME treatment showed increased c-PARP levels, particularly in A172 and T98G cells, with a time-dependent decrease in total PARP in U87 MG and A172 cells indicative of apoptosis progression. * Asterisks indicate statistical significance compared to control (0 μg/mL or 0 h) (*p* < 0.05). Error bars represent the mean ± SD of three independent experiments.

Given the observed increase in the sub-G_1_ phase signal in cell cycle assays under high-dose and prolonged CF-ME treatment (Figure 2A,B), the study further investigated whether CF-ME induces apoptosis in glioma cells. U87 MG, A172, and T98G cells were exposed to different concentrations of CF-ME for 72 h (Figure 3A) or treated with 100 μg/mL CF-ME for 24, 48, and 72 h (Figure 3B).

Apoptosis was analyzed via flow cytometry using annexin V-FITC and PI double staining. The results demonstrated that CF-ME induced apoptosis in a dose- and time-dependent manner (Figure 3A,B). To elucidate the molecular mechanisms underlying apoptosis, we examined the expression of apoptosis-related proteins, including cleaved caspase-3 (c-caspase-3) and cleaved PARP (c-PARP), using Western blotting. U87 MG, A172, and T98G cells were treated with 100 μg/mL CF-ME for varying durations (8, 16, 24, 48, and 72 h). CF-ME treatment increased the expression of c-caspase-3 without significant changes in total caspase-3 levels in A172 and T98G cells, while U87 MG cells showed decreased caspase-3 expression (Figure 3C). Similarly, c-PARP levels were increased in A172 and T98G cells, although no significant changes in pro-PARP levels were observed in T98G cells. In contrast, pro-PARP levels decreased in a time-dependent manner in U87 MG and A172 cells (Figure 3D). To further confirm apoptosis, a Caspase Colorimetric Activity Assay was performed to measure caspase-3 activity after CF-ME treatment (Table 2).

These results indicate that CF-ME activates these caspases, suggesting that CF-ME induces apoptosis by activating the caspase cascade and subsequent cleavage of PARP.

### 3.4. CF-ME Induces Autophagy in Glioma Cells

**Figure 4:**
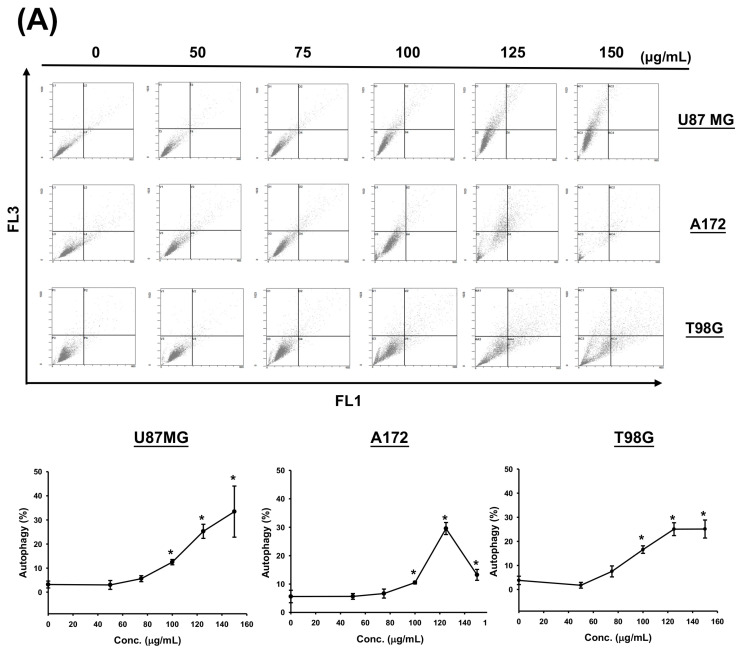

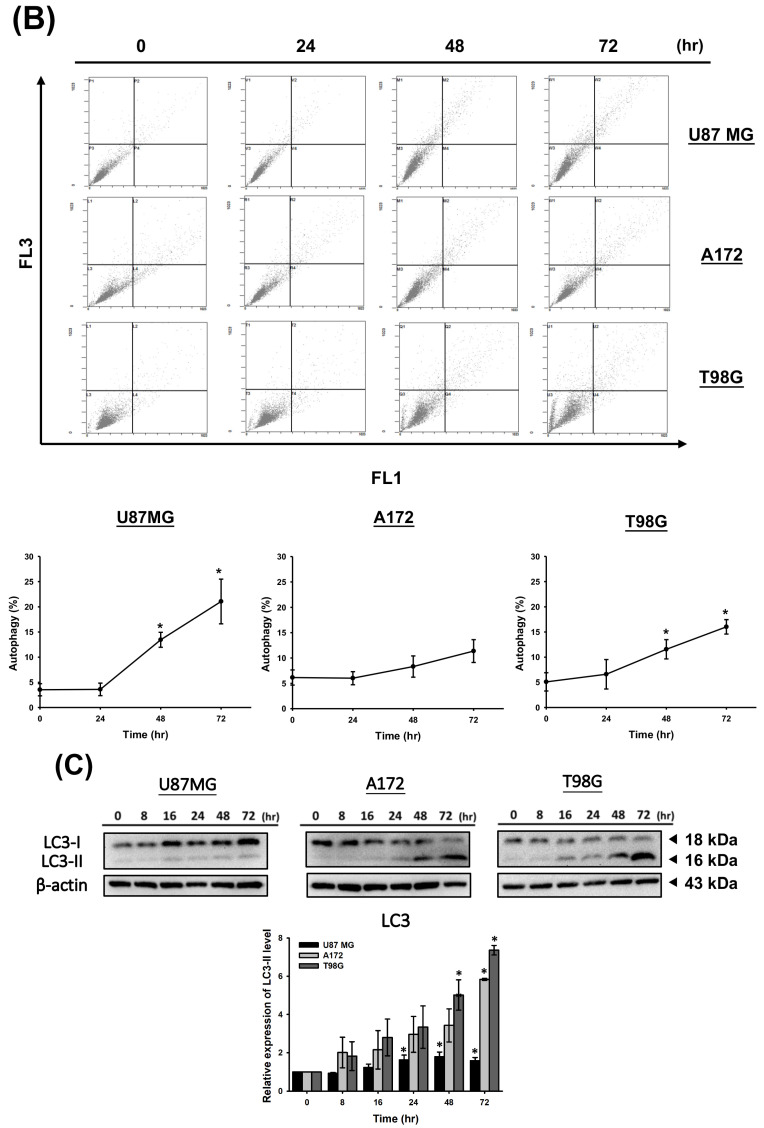
CF-ME induces autophagy in GBM Cells. (**A**) Flow cytometry analysis of autophagy in U87 MG, A172, and T98G GBM cell lines treated with CF-ME (0–150 μg/mL) for 72 h. Cells were stained with acridine orange to detect acidic vesicular organelles (AVOs), a hallmark of autophagy. The dot plots show a dose-dependent increase in AVO formation, indicating that CF-ME induces autophagy across all three cell lines. (**B**) Time-course analysis of autophagy in U87 MG, A172, and T98G cells treated with 100 μg/mL CF-ME for 24, 48, and 72 h reveals a time-dependent increase in AVO formation, with significant autophagic activity observed as early as 24 h, continuing to rise through 72 h. (**C**) Western blot analysis of LC3-I and LC3-II, markers of autophagy, in U87 MG, A172, and T98G cells treated with 100 μg/mL CF-ME for 0–72 h. The graphs below depict the relative expression levels of LC3-II normalized to β-actin. CF-ME treatment led to a time-dependent increase in LC3-II levels, confirming the induction of autophagy in glioma cells by CF-ME. * Asterisks indicate statistical significance compared to control (0 μg/mL or 0 h) (*p* < 0.05). Error bars represent the mean ± SD of three independent experiments.

To determine whether CF-ME induces autophagy in glioma cells, we examined the formation of acidic vesicular organelles (AVOs) and the expression of microtubule-associated protein light chain 3 II (LC3-II). U87 MG, A172, and T98G cells were treated with various concentrations of CF-ME for 72 h (Figure 4A) or with 100 μg/mL CF-ME for 24, 48, and 72 h (Figure 4B). Flow cytometry using acridine orange (AO) staining showed that CF-ME induced AVO formation in a dose- and time-dependent manner (Figure 4A,B). Western blotting showed increased LC3-II levels in U87 MG, A172, and T98G cells treated with 100 μg/mL CF-ME 8, 16, 24, 48, and 72 h (Figure 4C). These results indicate that CF-ME effectively induces autophagy in glioma cells.

### 3.5. CF-ME Suppresses Metastasis in Glioma Cells

**Figure 5:**
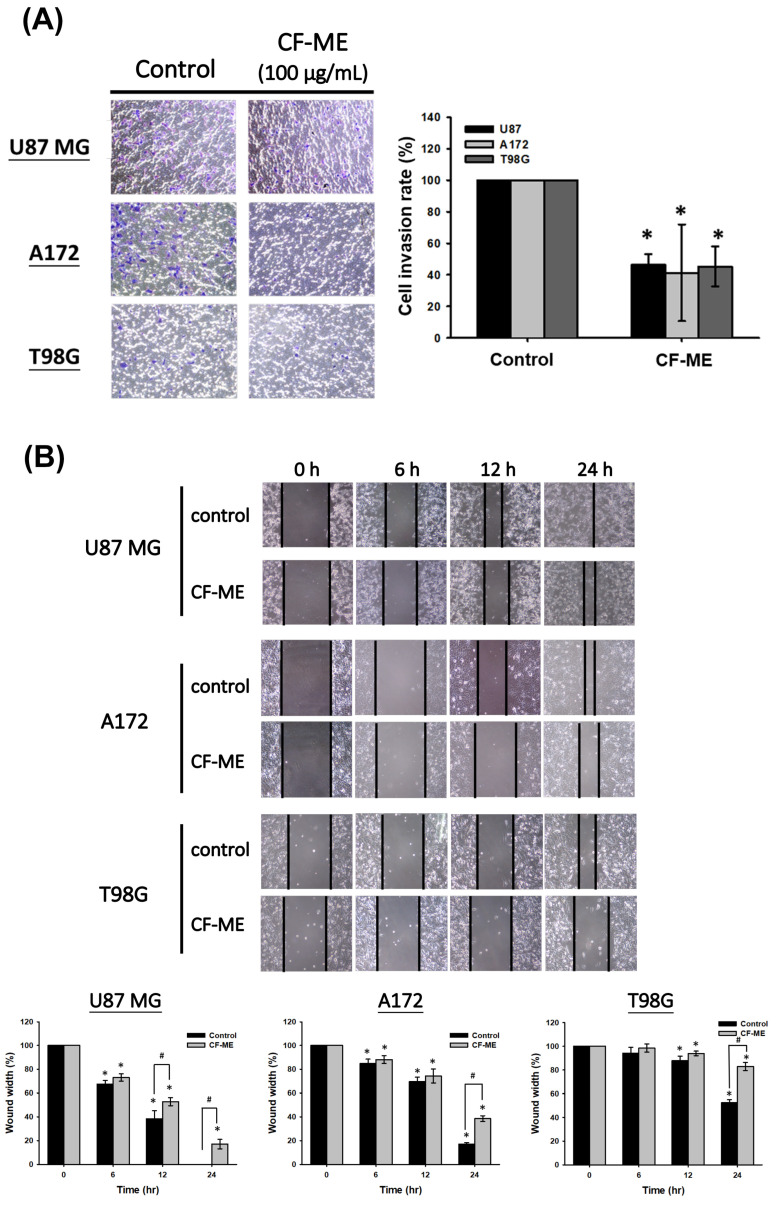

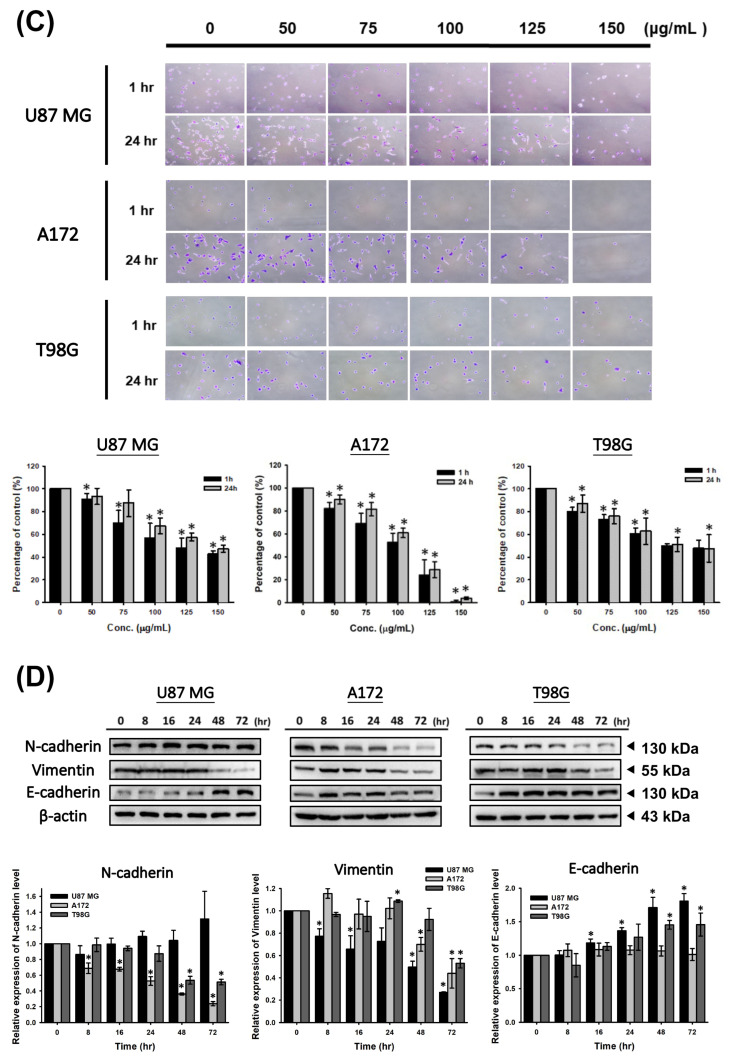
CF-ME inhibits invasion, migration, and adhesion of GBM Cells. (**A**) Invasion assay: U87 MG, A172, and T98G GBM cell lines were treated with 100 μg/mL CF-ME and subjected to a Transwell invasion assay. Representative images show the number of cells that invaded through the Matrigel-coated membrane compared to the control group. The bar graph quantifies the invasion rate as a percentage relative to the control, demonstrating that CF-ME significantly reduces the invasive capability of GBM cells. (**B**) Migration assay: Wound-healing assays were performed on U87 MG, A172, and T98G cells treated with 100 μg/mL CF-ME. Images were captured at 0, 6, 12, and 24 h post-scratch to assess cell migration into the wound area. The graphs quantify wound closure over time, showing that CF-ME significantly inhibits cell migration compared to control. (**C**) Adhesion assay: U87 MG, A172, and T98G cells were treated with CF-ME (0–150 μg/mL) and re-seeded for 1 h and 24 h to assess cell adhesion to the substrate. Representative images display the number of adherent cells at each concentration and time point. The bar graphs show that CF-ME treatment leads to a dose-dependent reduction in cell adhesion, with significant effects observed at higher concentrations and longer exposure times. (**D**) Western blot analysis: N-cadherin, vimentin, and E-cadherin, markers of epithelial–mesenchymal transition (EMT), were analyzed in U87 MG, A172, and T98G cells treated with 100 μg/mL CF-ME for 0–72 h. The graphs below depict the relative expression levels of N-cadherin, vimentin, and E-cadherin normalized to β-actin. * Asterisks indicate statistical significance compared to control is indicated by asterisks (*p* < 0.05), and hashtags indicate statistical significance between control and CF-ME in each treatment time point (# *p* < 0.05). Error bars represent the mean ± SD of three independent experiments.

To investigate the CF-ME inhibitory effect on glioma cell metastasis, we conducted Transwell, wound healing, and adhesion assays. First, U87 MG, A172, and T98G cells were treated with 100 μg/mL CF-ME for 72 h, then re-seeded in Transwell chambers (1 × 10^4^ cells/well) and incubated for 24 h. The Transwell assay results (Figure 5A) demonstrated reduced cell invasion in the lower chamber, indicating a CF-ME inhibitory effect. The wound-healing assay assessed cell migration. Scratched U87 MG, A172, and T98G cells treated with 100 μg/mL CF-ME were observed at 6, 12, and 24 h post-treatment. CF-ME-treated groups exhibited wider wound widths than the control, indicating suppressed glioma cell migration (Figure 5B). Finally, adhesion assays evaluated cells’ attachment ability. U87 MG, A172, and T98G cells treated with 0–150 μg/mL CF-ME for 72 h were re-seeded on 12-well plates (1 × 10^4^ cells/well) for 1 h and 24 h. The results (Figure 5C) showed a dose-dependent decrease in attached cells, suggesting CF-ME inhibition of glioma cell adhesion. Given CF-ME’s metastasis-suppressing ability, we explored its effect on epithelial–mesenchymal transition (EMT), a crucial process in cancer metastasis. EMT marker proteins—E-cadherin (epithelial), N-cadherin, and vimentin (mesenchymal)—were analyzed via Western blotting following CF-ME treatment (100 μg/mL) for 8, 16, 24, 48, and 72 h (Figure 5D). CF-ME treatment decreased N-cadherin and vimentin expression in glioma cells while increasing E-cadherin levels. However, N-cadherin expression remained unchanged in U87 MG cells (Figure 5D). These results suggest that CF-ME inhibits glioma cell metastasis by modulating EMT through the downregulation of mesenchymal markers and upregulation of epithelial markers.

### 3.6. CF-ME as a Potential Adjuvant Therapy for TMZ

**Figure 6:**
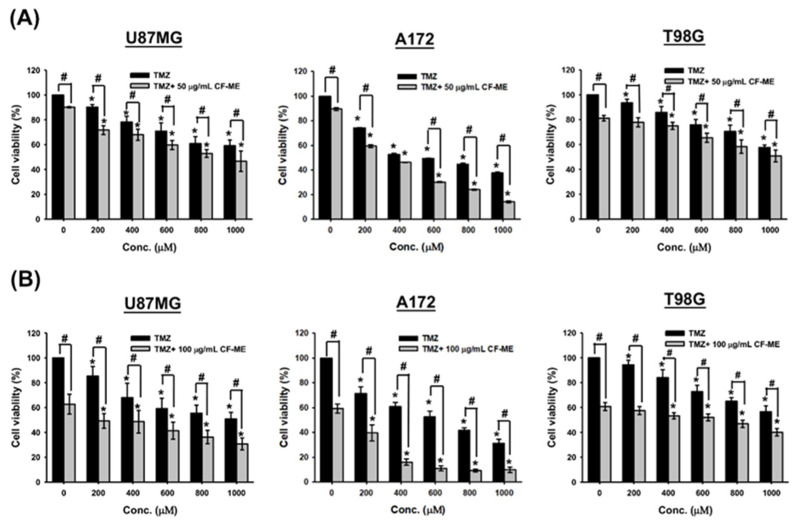
Effects of CF-ME in combination with TMZ on the viability of GBM cells. (**A**) Cell viability of U87 MG, A172, and T98G GBM cell lines treated with temozolomide (TMZ) alone or in combination with 50 μg/mL CF-ME was assessed using the MTT assay. Cells were exposed to varying concentrations of TMZ (0–1000 μM) for 72 h. The combination of CF-ME with TMZ significantly reduced cell viability across all concentrations compared to TMZ treatment alone, suggesting a synergistic effect of CF-ME in enhancing the cytotoxicity of TMZ. (**B**) Cell viability of U87 MG, A172, and T98G GBM cell lines treated with TMZ alone or in combination with 100 μg/mL CF-ME. The combination of CF-ME with TMZ at this higher concentration further reduced cell viability compared to TMZ alone, demonstrating that increasing the dose of CF-ME amplifies its synergistic effect with TMZ. * Asterisks indicate statistical significance compared to 0 μM TMZ is indicated by asterisks (*p* < 0.05), and hashtags indicate statistical significance between TMZ alone and TMZ combined with CF-ME (# *p* < 0.05). Error bars represent the mean ± SD of three independent experiments.

Glioma cells were pre-treated with low doses of CF-ME (50 μg/mL and 100 μg/mL) for 1 h, followed by exposure to varying concentrations of TMZ for 72 h. Cell viability was assessed using the MTT assay (Figure 6).

The combination of CF-ME and TMZ further suppressed glioma cell viability compared to TMZ alone (Figure 6). IC_50_ values of TMZ, both alone and in combination with 50 μg/mL and 100 μg/mL CF-ME, were calculated based on cell viability data. For U87 MG cells, the IC_50_ values were 911.1 ± 153.7 μM for TMZ alone, 784.8 ± 81.5 μM when combined with 50 μg/mL CF-ME, and 530.5 ± 87.4 μM when combined with 100 μg/mL CF-ME. For A172 cells, the IC_50_ values were 679.6 ± 13.9 μM for TMZ alone, 488.5 ± 33.8 μM when combined with 50 μg/mL CF-ME, and 259.7 ± 33.5 μM when combined with 100 μg/mL CF-ME. For T98G cells, the IC_50_ values were >1000 μM for TMZ alone, 979.8 ± 70.2 μM when combined with 50 μg/mL CF-ME, and 671.0 ± 45.8 μM when combined with 100 μg/mL CF-ME (Table 3).

These results suggest that combining CF-ME with TMZ could reduce the required dose of TMZ to achieve the same therapeutic effect, potentially mitigating the side effects of some drugs and overcoming drug resistance in glioma cells. Therefore, CF-ME shows promise as a potential adjuvant therapy to enhance the efficacy of TMZ in the treatment of glioma.

### 3.7. Content Analysis of Methanol Extract of C. rhizoma

**Figure 7:**
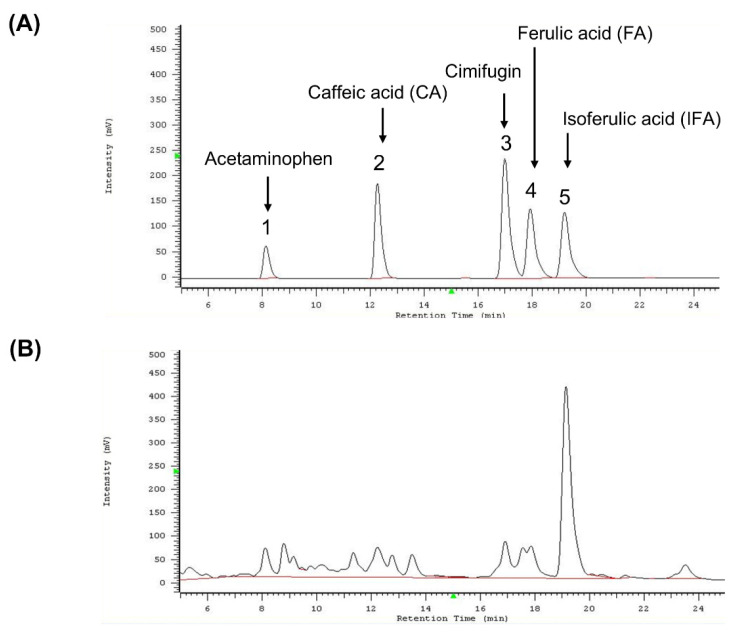
HPLC analysis of standard compounds and methanol extract of *Cimicifuga foetida*. (**A**) HPLC chromatogram showing the retention times of the internal standard acetaminophen and the standard compounds caffeic acid (CA), cimifugin, ferulic acid (FA), and isoferulic acid (IFA). The retention times were as follows: acetaminophen (~8.12 min), caffeic acid (~12.23 min), cimifugin (~16.91 min), ferulic acid (~17.85 min), and isoferulic acid (~19.13 min). (**B**) HPLC chromatogram of the methanol extract of *Cimicifuga foetida*. The chromatogram shows the positions of the identified index compounds (caffeic acid, cimifugin, ferulic acid, and isoferulic acid) after comparison with the retention times of the standard compounds.

To verify the species of *C. rhizoma* used in this study, key compounds in the methanol extract were analyzed using high-performance liquid chromatography (HPLC). The retention times were 8.12 min for acetaminophen (internal control), 12.23 min for caffeic acid, 16.91 min for cimifugin, 17.85 min for ferulic acid, and 19.13 min for isoferulic acid (Figure 7).

These retention times were compared with previously published data to identify the species. Among the three species of *C. rhizoma* listed in the Taiwan Herbal Pharmacopeia (*C. foetida*, *C. heracleifolia*, and *C. dahurica*), only *C. foetida* contained all four index compounds. Thus, the plant material was confirmed to be *C. foetida*. To quantify the content of these compounds in the methanol extract, standard curves (3.125–25 μg/mL) were generated for caffeic acid, cimifugin, ferulic acid, and isoferulic acid (Table 1). The methanolic extract contained 0.11% caffeic acid, 0.08% cimifugin, 0.11% ferulic acid, and 0.78% isoferulic acid (Table 4). These findings validated the plant species and provided a detailed chemical profile essential for experimental reproducibility.

### 3.8. Investigation of the Anti-Glioma Effects of Index Compounds in CF-ME

**Figure 8:**
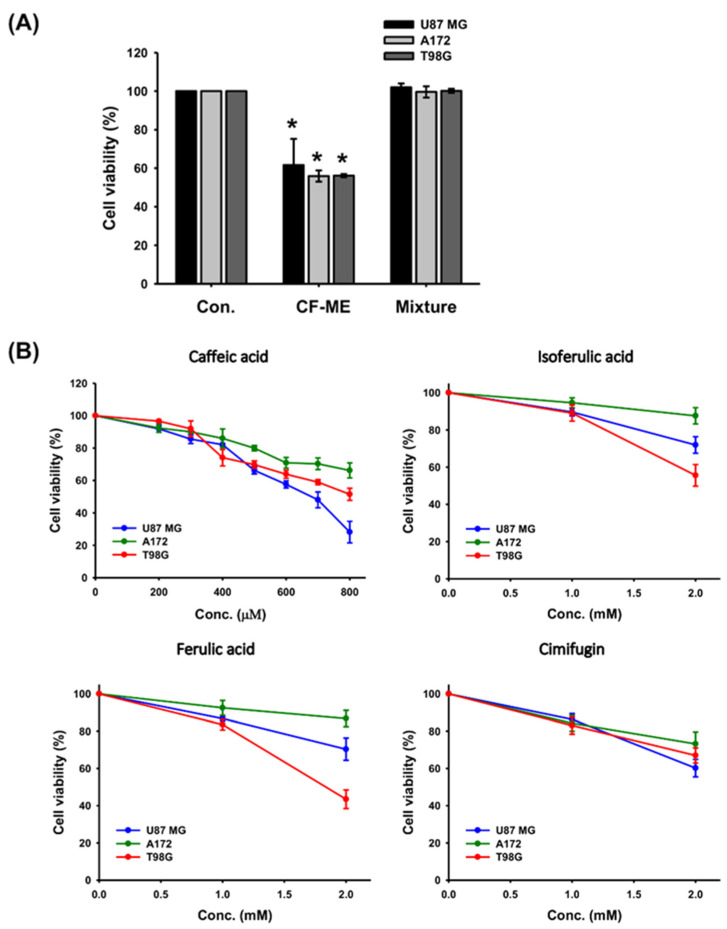
Effect of individual and combined index compounds from CF-ME on the viability of GBM cells. (**A**) Cell viability of U87 MG, A172, and T98G GBM cell lines after 72 h of treatment with CF-ME and a mixture of index compounds (caffeic acid, isoferulic acid, ferulic acid, and cimifugin) at concentrations equivalent to those in 100 μg/mL of CF-ME, as determined via HPLC analysis. The results showed that while CF-ME significantly reduced cell viability in all three GBM cell lines, the mixture of index compounds did not have the same effect. (**B**) Dose-response curves showing the effect of individual index compounds—caffeic acid, isoferulic acid, ferulic acid, and cimifugin—on the viability of U87 MG, A172, and T98G GBM cell lines. Cells were treated for 72 h with increasing concentrations of each compound. The results indicate that each compound has a slight inhibitory effect on cell viability, with caffeic acid being more effective at higher concentrations compared to the other compounds. * Asterisks indicate statistical significance compared to the control group (*p* < 0.05). Error bars represent the mean ± SD of three independent experiments.

Previous findings have confirmed the in vitro anti-glioma activity of CF-ME. To identify active components, we assessed a mixture of the four index compounds—caffeic acid, cimifugin, ferulic acid, and isoferulic acid—at a concentration equivalent to 100 μg/mL of CF-ME, using the MTT assay. Unexpectedly, this mixture exhibited minimal anti-glioma activity (Figure 8A). Further investigation using the MTT assay of individual compounds at higher doses (up to 800 μM) showed that caffeic acid at 800 μM reduced the cell viability of U87 MG cells to below 50%, but it had a limited effect on A172 and T98G cells, with their viability remaining above 50%. Ferulic acid, isoferulic acid, and cimifugin at higher doses (1–2 mM) slightly inhibited the viability of all three glioma cell lines (Figure 8B). These results suggested that the individual index compounds did not account for the full anti-glioma effects of CF-ME. Further research is necessary to identify the specific active compounds or potential synergistic interactions within CF-ME that contribute to its anti-glioma activity.

## 4. Discussion

This study systematically evaluated the effects of water (CF-WE) and methanol (CF-ME) extracts on glioma cell growth and viability, focusing on the latter due to its demonstrated efficacy. *Cimicifuga*, a genus encompassing over 18 species, is traditionally used in various cultures [18]. According to the Taiwan Herbal Pharmacopeia (4th edition), Taiwan utilizes *C. foetida*, *C. heracleifolia*, and *C. dahurica*, each with distinct chemical profiles [9]. HPLC fingerprinting is commonly employed to confirm *Cimicifuga* species based on the presence of key compounds, including phenolic acids, triterpenes, and chromones. Specifically, the phenolic acids caffeic acid, ferulic acid, and isoferulic acid, as well as the chromone cimifugin, have been used as marker compounds to identify the botanical origin of *Cimicifuga* herbs [10,21,22]. According to He et al., only *C. foetida* contains all four marker compounds [21]. In the current study, HPLC fingerprinting confirmed that the use of *C. foetida* in our methanol extract (CF-ME) identified caffeic acid, ferulic acid, isoferulic acid, and cimifugin at concentrations of 0.11%, 0.11%, 0.78%, and 0.08%, respectively, with the isoferulic acid content exceeding 0.1%. These findings further revealed that CF-ME significantly inhibited glioma cell growth, inducing G_1_ phase cell cycle arrest, apoptosis, and autophagy and suppressing metastasis. These results suggest CF-ME as a potential therapeutic agent for glioma treatment, particularly in combination with TMZ.

CF-ME induced G_1_ phase cell cycle arrest in glioma cells, potentially mediated by upregulation of GADD45A and p21 and downregulation of CDK6. GADD45A is known to regulate cell survival and apoptosis by inducing G1 and G2/M cell cycle arrest or by activating the MAPK pathways [23]. Previous studies have shown that overexpression of GADD45A can cause G_2_/M cell cycle arrest and reduce proliferation in T24 bladder cancer cells, as well as induce G_0_/G_1_ and G_2_/M phase arrest in EBV^+^ B lymphoma cells through the activation of the p38 MAPK/TAp73/GADD45A axis [24]. Additionally, GADD45A has been implicated in the regulation of apoptosis via p53 activation and induces apoptosis in pancreatic and rectal cancer cells via MAPK signaling. This mechanism of action is crucial, as it halts cell proliferation, thereby limiting tumor growth. The role of GADD45A in inducing p21 further supports this, as p21 contributes to cell cycle arrest by inhibiting cyclin-dependent kinases like CDK6 [25]. For instance, in liver cancer cells, the activation of GADD45A by isocorydine derivatives upregulates p21, which causes G2/M phase arrest and suppresses tumor growth [25]. Beyond its role in cell cycle arrest, CF-ME has been shown to induce apoptosis in glioma cells, likely through the activation of the caspase cascade and PARP cleavage, both of which are well-established pathways in programmed cell death. The dual action of CF-ME—inducing cell cycle arrest and promoting apoptosis—significantly enhances its anticancer effects, positioning it as a promising candidate for glioma treatment. Additionally, previous research on *Cimicifuga* species has shown that extracts from *C. dahurica* can inhibit breast cancer cell growth. Compounds such as caffeic acid and ferulic acid found in CF-ME have demonstrated anticancer activities in various cancer cell lines by inducing ROS production, disrupting mitochondrial membrane potential, and triggering apoptosis [26,27,28,29].

CF-ME induced not only apoptosis but also autophagy in glioma cells, a process that can either promote cell survival or lead to cell death, depending on the context. Apoptosis, characterized by the activation of the caspase cascade and PARP cleavage, is a well-established mechanism of programmed cell death [30]. CF-ME’s ability to activate this pathway significantly contributes to its anticancer effects in glioma cells. In addition, the upregulation of LC3-II, a marker of autophagy, suggests that CF-ME also activates the autophagic pathway. While autophagy is typically a protective mechanism under stress, it can also lead to autophagic cell death when excessively activated. The dual induction of apoptosis and autophagy by CF-ME adds complexity to its anti-glioma activity, potentially enhancing its therapeutic efficacy.

This study demonstrated CF-ME’s ability to suppress glioma cell metastasis by inhibiting cell invasion, migration, and adhesion. Metastasis, a critical factor in cancer progression, involves the spread of cancer cells from the primary site to distant sites. A key mechanism in metastasis is the EMT, which plays a vital role in tumor formation, malignancy, and cell migration. In GBM, EMT contributes to the cancer’s aggressive invasiveness. Cells in an epithelial state exhibit strong adhesion, while those in a mesenchymal state have enhanced migratory abilities. The study found that CF-ME exerts its anti-metastatic effects by modulating EMT markers, specifically decreasing mesenchymal markers (N-cadherin and vimentin) and increasing the epithelial marker (E-cadherin). EMT-inducing transcription factors (EMT-TFs), such as ZEB, Snail, and Twist, facilitate cancer cell invasion by activating mesenchymal and suppressing epithelial markers. Previous studies have shown that drugs can inhibit metastasis by regulating these EMT-related molecules, as seen with polydatin and sotetsuflavone, in other cancer types. These findings suggest that CF-ME may effectively reduce tumor spread by targeting EMT in GBM.

TMZ is a lipophilic small molecule (194 Da) and an oral imidazotetrazine alkylating agent that can cross the blood–brain barrier, making it a key treatment for glioblastoma [7,31]. Its cytotoxic effects stem from O6-methylguanine formation, leading to mismatches with thymine during DNA replication. This mismatch triggers a futile cycle in the mismatch repair system, ultimately resulting in DNA damage and cell death [7]. Although TMZ treatment often induces G_2_-M phase arrest in glioma cells, it leads to apoptosis only in a subset of treated cells [32]. This study found that the combination of CF-ME and TMZ further reduced glioma cell viability, suggesting a synergistic effect. This synergy could potentially reduce TMZ dosage, thereby minimizing its side effects and overcoming drug resistance. These findings suggest that CF-ME is a promising adjuvant therapy for glioma with the potential to improve patient outcomes by enhancing the efficacy of TMZ treatment.

While this study provides valuable insights, the findings have broader implications for cancer treatment and patient outcomes. The ability of CF-ME to enhance the efficacy of TMZ, induce apoptosis and autophagy, and suppress metastasis suggests its potential to improve the therapeutic landscape for glioblastoma. CF-ME as an adjuvant therapy could reduce the required dosage of TMZ, mitigating side effects such as hematological toxicity and liver damage while maintaining or enhancing therapeutic efficacy. This is particularly relevant for patients who develop resistance to TMZ, as CF-ME’s multi-targeted approach could offer a new avenue for overcoming treatment barriers. Furthermore, CF-ME’s modulation of EMT markers and inhibition of metastasis may hold significant potential for preventing the aggressive spread of glioblastoma cells, thereby improving both treatment efficacy and patient survival rates. To move these findings from in vitro to in vivo applications, several key steps are required. First, studies using animal models are essential to validate CF-ME’s efficacy, pharmacokinetics, and toxicity profile. These studies will help determine the optimal dosage, possible side effects, and interactions with existing treatments like TMZ. Upon successful preclinical testing, clinical trials could be initiated to evaluate CF-ME’s impact on patient outcomes in a clinical setting, ensuring its effectiveness and safety in humans. This transition is critical for establishing CF-ME as a viable option in glioma therapy. However, this study has several limitations. First, the research was conducted using in vitro glioblastoma cell lines, which, while widely used for preliminary assessments of anticancer activity, do not fully replicate the complex tumor microenvironment found in vivo. As a result, the observed effects of CF-ME and its constituents may vary in animal models or clinical settings. Second, although the study identified several active pathways, including apoptosis, autophagy, and cell cycle arrest, the exact molecular targets and mechanisms remain unclear. Further studies are necessary to elucidate these mechanisms in detail and determine whether the effects are due to specific protein interactions or broader cellular stress responses. Additionally, when tested separately, the individual compounds in CF-ME exhibited only slight inhibitory effects on glioblastoma cell viability, suggesting that CF-ME’s anti-glioma activity may result from synergistic interactions among its multiple constituents. However, the nature of these potential synergies was not explored in this study, and future research should focus on identifying the most effective combinations of compounds and testing the BBB permeability of these individual active compounds. Finally, the study did not address the pharmacokinetics, bioavailability, or potential toxicity of CF-ME or its individual components in vivo, which are critical factors in determining the therapeutic potential of any compound. Without these data, the immediate applicability of these findings to clinical contexts remains limited. Future studies should aim to fully characterize CF-ME’s therapeutic efficacy and safety, including its effects on normal tissues and its potential interactions with standard glioma treatments like TMZ. Comprehensive research will be essential to translate these promising in vitro results into clinically viable treatments for glioma.

## 5. Conclusions

CF-ME exhibits potent anti-glioma activity through multiple mechanisms, including G1 phase cell cycle arrest, apoptosis, autophagy, and metastasis inhibition. CF-ME’s ability to enhance the efficacy of TMZ further underscores its potential as an adjuvant therapy for gliomas. However, the individual index compounds identified in CF-ME did not fully account for its effects, suggesting potential synergistic actions among its multiple constituents. Future studies are warranted to isolate and identify these active compounds, to elucidate their interactions, and to explore their pharmacokinetics and bioavailability in vivo. Additionally, in-depth investigations into the safety, efficacy, and potential clinical applications of CF-ME are essential to fully realize its therapeutic potential. Overall, CF-ME stands out as a promising candidate for further development in glioma treatment, but comprehensive research is needed to translate these findings into clinical settings.

## Figures and Tables

**Table 1:** IC_50_ values of malignant glioma cells and normal glial cells after treatment with extract of *Cimicifuga foetida*. U87 MG, A172, T98G, and SVGp12 cells were treated with different doses of CF-WE and CF-ME for 72 h, respectively. Cell viability was detected using an MTT assay with an ELISA reader to measure absorbance values. IC_50_ values were calculated after plotting the regression lines. Data are presented as means ± standard deviation.

Cell Lines	IC_50_ (μg/mL)	
	CF-WE	CF-ME
U87 MG	662.5 ± 70.0	94.3 ± 5.2
A172	>800	96.5 ± 3.7
T98G	>800	97.3 ± 1.6
SVGp12	>800	>150

U87 MG, A172, and T98G were malignant glioma cell lines; SVGp12 was human normal glial cell line.

**Table 2:** Effect of CF-ME on the activities of caspase 3. U87 MG, A172, and T98G cells were treated with 100 μg/mL CF-ME for 0, 24, 48, and 72 h and extracted proteins. Activities of caspase 3 were determined using the Caspase Colorimetric Activity Assay Kit. Absorbance values were measured using an ELISA reader, and fold changes compared to the control group were calculated. Data are presented as means ± standard deviation (*n* = 3). * *p* < 0.05 indicates a statistically significant difference compared to the respective control groups.

Caspase	Cell Line	100 μg/mL CF-ME Treatment (h)			
		0	24	48	72
	U87 MG	1	1.7 ± 1.2	3.3 ± 1.5	4.3 ± 0.6 *
Caspase 3	A172	1	1.3 ± 0.6	3.2 ± 0.8 *	5.0 ± 1.6 *
	T98G	1	3.0 ± 1.0 *	4.7 ± 0.6 *	7.0 ± 1.0 *

**Table 3:** Combination of CF-ME with temozolomide (TMZ) could decrease the IC_50_ values of TMZ. U87 MG, A172, and T98G cells were treated with different doses of TMZ and combined with low doses (50 μg/mL and 100 μg/mL) of CF-ME for 72 h. Cell viability was detected using an MTT assay with an ELISA reader to measure absorbance values. IC_50_ values were calculated after plotting the regression lines. Data are presented as means ± standard deviation (*n* = 3). * *p* < 0.05 indicates a statistically significant difference compared to the respective control groups.

Cell Lines	IC_50_ (μM) of TMZ at Different Concentrations of CF-ME		
	0 μg/mL	50 μg/mL	100 μg/mL
U87 MG	911.1 ± 153.7	784.8 ± 81.5	530.5 ± 87.4 *
A172	679.6 ± 13.9	488.5 ± 33.8 *	259.7 ± 33.5 *
T98G	>1000	979.8 ± 70.2 *	671.0 ± 45.8 *

**Table 4:** Quantitative analysis of four index compounds in the methanol extracts of *Cimicifuga foetida* (CF-ME). 10 mg/mL of CF-ME was detected via HPLC. The data were compared with the standard curves of four index compounds to calculate their respective percentage content in the methanol extract.

Index Compound	Standard Curve	Content (%)
Caffeic acid	y = 0.1221x + 0.1536	0.11
Cimifugin	y = 0.1827x + 0.2071	0.08
Ferulic acid	y = 0.1189x + 0.1466	0.11
Isoferulic acid	y = 0.1144x + 0.1514	0.78
Index compound	Standard curve	Content (%)

## Data Availability

The original contributions presented in the study are included in the article, further inquiries can be directed to the corresponding author.
